# Open Availability of Patient Medical Photographs in Google Images Search Results: Cross-Sectional Study of Transgender Research

**DOI:** 10.2196/jmir.8787

**Published:** 2018-02-26

**Authors:** Zack Marshall, Fern Brunger, Vivian Welch, Shabnam Asghari, Chris Kaposy

**Affiliations:** ^1^ School of Social Work McGill University Montreal, QC Canada; ^2^ Division of Community Health & Humanities Memorial University of Newfoundland St John's, NL Canada; ^3^ Bruyere Research Institute Ottawa, ON Canada; ^4^ Department of Family Medicine Memorial University of Newfoundland St John's, NL Canada; ^5^ Center for Rural Health Studies Faculty of Medicine Memorial University of Newfoundland St John's, NL Canada

**Keywords:** informed consent, photography, open access publishing, confidentiality, image repositories, big data, publication ethics, transgender persons

## Abstract

**Background:**

This paper focuses on the collision of three factors: a growing emphasis on sharing research through open access publication, an increasing awareness of big data and its potential uses, and an engaged public interested in the privacy and confidentiality of their personal health information. One conceptual space where this collision is brought into sharp relief is with the open availability of patient medical photographs from peer-reviewed journal articles in the search results of online image databases such as Google Images.

**Objective:**

The aim of this study was to assess the availability of patient medical photographs from published journal articles in Google Images search results and the factors impacting this availability.

**Methods:**

We conducted a cross-sectional study using data from an evidence map of research with transgender, gender non-binary, and other gender diverse (trans) participants. For the original evidence map, a comprehensive search of 15 academic databases was developed in collaboration with a health sciences librarian. Initial search results produced 25,230 references after duplicates were removed. Eligibility criteria were established to include empirical research of any design that included trans participants or their personal information and that was published in English in peer-reviewed journals. We identified all articles published between 2008 and 2015 with medical photographs of trans participants. For each reference, images were individually numbered in order to track the total number of medical photographs. We used odds ratios (OR) to assess the association between availability of the clinical photograph on Google Images and the following factors: whether the article was openly available online (open access, Researchgate.net, or Academia.edu), whether the article included genital images, if the photographs were published in color, and whether the photographs were located on the journal article landing page.

**Results:**

We identified 94 articles with medical photographs of trans participants, including a total of 605 photographs. Of the 94 publications, 35 (37%) included at least one medical photograph that was found on Google Images. The ability to locate the article freely online contributes to the availability of at least one image from the article on Google Images (OR 2.99, 95% CI 1.20-7.45).

**Conclusions:**

This is the first study to document the existence of medical photographs from peer-reviewed journals appearing in Google Images search results. Almost all of the images we searched for included sensitive photographs of patient genitals, chests, or breasts. Given that it is unlikely that patients consented to sharing their personal health information in these ways, this constitutes a risk to patient privacy. Based on the impact of current practices, revisions to informed consent policies and guidelines are required.

## Introduction

This paper focuses on the collision of three factors: a growing emphasis on sharing research through open access publication, an increasing awareness of big data and its potential uses, and an engaged public interested in the privacy and confidentiality of their personal health information. One conceptual space where this collision is brought into sharp relief is the open availability of patient medical photographs from peer-reviewed journals in the search results of online image databases such as Google Images.

Researchers, funders, policy makers, and the public promote the importance of open access research publications [1]. In some instances, this perspective is communicated through public access mandates established by research institutions, funders, or governments [2]. Open access publications allow us to share information more widely with relevant stakeholders including the public, policy makers, and clinicians. Increased access to research supports the principles of accountability, replicability, transparency, and equity. Such access has the potential to reduce research waste and has been promoted as a core component of the Responsible Research and Innovation framework [3].

While open access publishing is increasingly being embraced, big data and access to massive online databases are expanding. Google Images was launched in 2001, and in its first year included 250 million images [4]. It is estimated that it now has over 1 trillion indexed images [5]. Google obtains its images from crawling websites [6] and indexing those images within its searchable database [7]. Given the way Google Images works, it is not surprising that photographs from peer-reviewed publications are available online. Physicians and their patients may not realize that sensitive medical photographs published in closed and open access publications are now also freely available within these image databases.

Clinical photographs form part of the patient medical record [8]. Some have argued that patients also own or at least co-own their medical images [9]. As such, it is important to attend to the ethical aspects of capturing, storing, transferring, and using these images [10]. Current guidelines underline the need for prior written consent from patients before taking clinical photographs, using them for teaching or research purposes, or publishing them in peer-reviewed journals, books, or pharmaceutical publications [11]. Similarly, some journals, including the *BMJ*, call for written consent from patients (and photographers) before publication (ie, Uses of Images policy) [12]. Within this context, clinicians are expected to balance the importance of medical photography for education, research, and clinical care, with the ethical principle of patient autonomy, including informed consent and confidentiality [9], and respect for patients.

Until recently, medical photography consent forms did not contain any reference to electronic publishing or social media (eg, [13]). With growing awareness of the potential for materials published electronically, clinicians are recommending enhanced attention to informed consent and greater specificity in consent forms [14]. New publications focusing on informed consent to use images highlight the ways information can be shared electronically and that photographs cannot later be removed if the patient removes consent [15]. Aside from general warnings, we were not able to locate examples of medical photography consent forms that include permission to publish clinical photographs on Google Images or that warn of this specific risk.

In many countries, transgender, gender non-binary, and other gender diverse (trans) people are required to work with clinicians in order to access medical transition. These mandatory interactions can create complex dynamics between patients and health care providers, including challenges related to voluntary and informed consent in the contexts of clinical research and medical photography. While the privacy and confidentiality of medical photographs are important for all patients, privacy breaches carry an increased possibility of harm for trans people. This risk exists because trans people experience heightened rates of discrimination, harassment, and violence [16,17], particularly those who are visibly gender non-binary or whose trans identities become known to individuals in their lives who may not have been previously aware of this information [18].

In this study, we identified a sample of peer-reviewed publications that included clinical photographs of trans people and searched for the publications and their associated photographs on Google Images. The objective of this study was to assess the availability of patient clinical photographs from published journal articles in Google Images search results and the factors impacting this availability. We hypothesized that if journal articles were openly available online, either through open access or article repositories, that the likelihood of finding the accompanying photographs in Google Images search results would be increased. The results of this study will inform privacy and informed consent guidelines in relation to the publication of medical photographs in peer-reviewed journals, including recommendations for clinicians and publishers.

## Methods

The sample for this cross-sectional study was identified from an evidence map of peer-reviewed empirical research including transgender, gender non-binary, and other gender diverse (trans) people. In order to develop the original dataset, a comprehensive search strategy was developed in collaboration with a health sciences librarian and included 220 trans-related search terms and 15 academic databases from the fields of health, education, social science, business, and the humanities. Further details about study identification and inclusion and exclusion criteria are found in the study protocol paper [19]. A sample search strategy is included in Multimedia Appendix 1.

**Figure 1:**
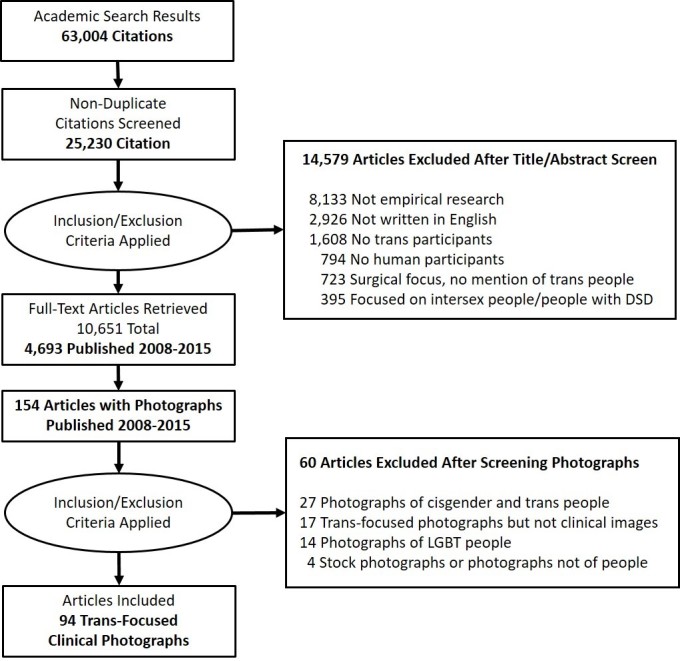
Preferred Reporting Items for Systematic Reviews and Meta-Analyses (PRISMA) flow diagram.

The inclusion criteria were designed to identify empirical research published in English in peer-reviewed journals that included trans participants. As noted in the Preferred Reporting Items for Systematic Reviews and Meta-Analyses (PRISMA) flow diagram in Figure 1, the initial search produced 25,230 references after duplicates were removed and 10,651 met the initial screening criteria; 4693 of these articles were published between 2008 and 2015. After screening on full text, 154 references published between 2008 and 2015 were identified that included photographs. We identified 94 articles with medical photographs of trans participants (see Multimedia Appendix 2 for a full list of this sample). For each reference, images were individually numbered in order to track a total of 605 medical photographs. We excluded 60 references for the following reasons: (1) the article included images of both cisgender and transgender people (27 references), (2) the article was trans-focused but the photographs were not clinical (17 references), (3) the article included images of lesbian, gay, bisexual, or transgender (LGBT) people (14 references), and (4) the article included only stock images or photographs that were not of people (4 references).

### Google Images Search

For each publication, the first author (ZM) searched for the reference in Google Images using the full title of the article in quotation marks. This approach was selected after piloting several different strategies including searching for the full title without quotation marks, searching for the last name of the first author and the first few words of the title, or the last name of the first author and keywords in the title. The aim was to find an approach that was consistent, feasible, and that could be easily replicated. Searching for the title in quotation marks produced the most focused results, typically including 2-4 pages of images.

The Tor Browser is “a proxy that masks the location information and browsing history of the user, allowing for anonymous use of the Internet” (p. 18) [20]. This browser was used to conduct searches in order to minimize the influence of Google Analytics. For each individual search, a new identity was established within the Tor Browser and the Internet Protocol (IP) address for the last location in the Tor circuit was documented. Results for each search were saved in PDF. Any images identified from the search were compared to the photographs in the original article. For each image that was a match, we saved an electronic copy of the photograph. In addition, we clicked the hyperlink to “Visit Webpage” in order to determine the original source of the photograph. Results from the source webpage were also saved as PDFs.

One of the reasons for saving the searches as PDFs is that the results of Google Images searches are not static and potentially they will change over time. For this reason, it was important to have clear documentation of search results and images that were located as part of the search. Details of each search were saved in an Microsoft Excel spreadsheet including the last name of the study’s first author, image identification number, URL for the image, URL for the source webpage, IP address from the Tor Browser, search date, and details about whether the image was found or not. All searches were conducted between May 21 and June 23, 2017.

### Online Availability Search

As part of the data collection process, we were also interested in knowing whether the publication was available through open access and if this might influence whether the photographs were located on Google Images. A strategy for checking whether references were available through open access was developed with the team’s health sciences librarian. One reviewer coded 10% of the trans-focused studies. A second reviewer verified the data extracted. Based on this information, we took two steps to check for open access using Google Scholar, PubMed, Researchgate.net, and Academia.edu. We searched the title of the article in quotation marks using Google Scholar. If the article was listed, we clicked through to either the paywall [21] or to the PDF.

Because some articles are available through the National Library of Medicine but are not available through Google Scholar (eg, *Journal of American Public Health Association*), we also searched for all references in PubMed. In addition, we searched for each reference on Researchgate.net and on Academia.edu, two social networking sites where researchers can share preprints and PDFs of academic publications. While Google Scholar often includes information about whether a PDF is available on Researchgate.net or Academia.edu, this is not always the case.

### Dataset Demographics

In order to characterize the data at the level of individual photographs, we identified the following information for each photograph: (1) body location: face, chest, genitals, skin graft site, other, (2) population: trans women (including male-to-female transsexuals and people on the trans feminine spectrum), trans men (including female-to-male transsexuals and people on the trans masculine spectrum), and other, (3) timing: presurgery or pretreatment (including preoperative), during surgery or treatment, postsurgery or posttreatment (including immediately postoperative), specimen, autopsy, and other, (4) anonymization: anonymized, not anonymized, or not applicable, and (5) whether the image was in color or black and white. Images that were not photographs (such as X-rays and magnetic resonance images) were not included in this sample. Although such images may constitute personal health information, they are often considered separate from photographs. For clarity, we maintained a focus on medical photographs.

### Patient Involvement

This study sits within a larger project focused on the development of an evidence map documenting research with trans individuals and communities. As part of the process of constructing evidence maps, it is recommended that researchers clarify concepts and engage key stakeholders in considering the potential scope of the review [22]. Accordingly, individual consultations were held with members of trans and cisgender communities to discuss terminology, search scope, and potential uses of study results. In addition, people from sexual and gender diverse communities were hired as research assistants on the project when possible. This specific project on the inclusion of medical photographs of trans patients in online image repository search results did not involve consultation with patients or trans community members. There will be an emphasis on patient and community involvement during the dissemination phase, including presentations at relevant trans health conferences and community events.

## Results

### Google Images Availability

In total, 37% (35/94) of articles included at least one photograph that was found on Google Images. When we searched the photographs individually, 20.3% (123/605) of clinical photographs in the articles were found on Google Images.

### Online Availability

Over a third (38% [36/94]) of the references were available through open access on the journal website, through the journal publishers, or through PubMed. In addition, we checked for availability of articles on other websites including Researchgate.net, Academic.edu, and institutional repositories. Over half of the references (59% [55/94]) were available through at least one of these channels, meaning that article content was freely accessible.

### Dataset Demographics

**Figure 2:**
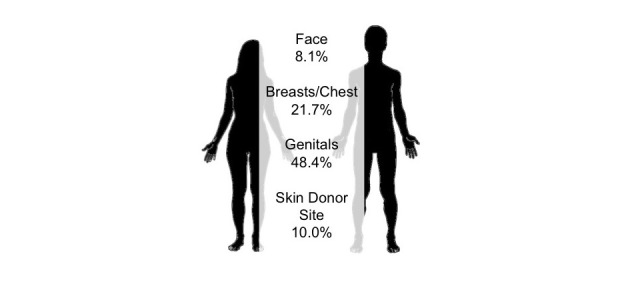
Characteristics of medical photographs.

The average publication included 6 photographs, with a range of 1-29 images. In total, 605 photographs were included in the sample. The information presented in this section relates to analysis at the level of individual photographs. Photographs most commonly included genitals (48.4% [293/605]), chest or breasts (21.7% [131/605]), skin donor site (primarily forearm or leg) (10.1% [61/605]), and face (8.1% [49/605]) (see Figure 2).

According to descriptions provided, 63.8% (386/605) of the individual photographs were of trans men or female-to-male trans people, 35.2% (213/605) were of trans women or male-to-female trans people, and 1% (6/605) could not be easily categorized. In terms of timing, 22.0% (133/605) of images were taken before treatment or surgery, 32.0% (194/605) of images were taken during surgery, and 37.4% (226/605) were taken postsurgery or after treatment. In addition, 3.1% (19/605) of images were of specimens, and 3.3% (20/605) were autopsy photographs. The majority (80.0% [484/605]) of the images were printed in color, and a fifth (20.0% [121/605]) in black and white.

Nearly 10% (58/605) of the images included identifiable elements including faces, full-body autopsy images, or potentially recognizable tattoos. Authors made anonymization attempts in almost a quarter of these photographs (24% [14/58]). This included placing small black boxes [23,24] or black bars [25] over the person’s eyes. In one case, the authors taped a large piece of paper over the image covering the person’s nose, eyes, and forehead [26]. For three-quarters (74% [43/58]) of the photographs with identifiable elements, there were no attempts at anonymization.

### Documenting Informed Consent

We reviewed all 94 papers to verify if any mention was made of informed consent. Seven papers described obtaining written informed consent for publication of medical images from trans patients. For example, Aminsharifi et al [27] made the following statement, “Written informed consent was obtained from the patient for the publication of this case and accompanying images. A copy of the written consent is available for review by the Editor-in-Chief of this journal on request” (p. 268). We did not write to individual authors to confirm whether they obtained informed consent to publish clinical photographs and whether the consent form included explicit mention of electronic publishing or social media.

### Factors Affecting Google Images Availability

**Figure 3:**
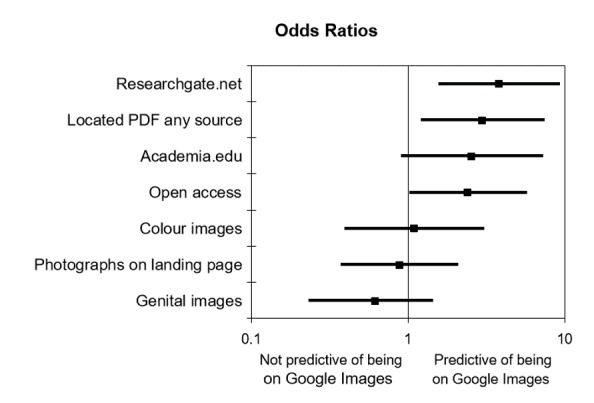
Odds ratios indicating likelihood of clinical photographs being included in Google Images search results.

In order to understand what factors might be contributing to the number of clinical photographs available on Google Images, we calculated the odds ratios (OR) of availability on Google Images across specific factors that we expected might play a role based on the belief that Google may be filtering images of genitals from their search results. We also felt that article availability on Web-based platforms could influence whether photographs were included in Google Images search results. In addition, because of the ways Google crawls websites, we thought that if photographs were visible from the original landing page [28], then they might be more likely to appear in search results. As noted in Figure 3, the ability to locate a PDF of the article online through open access, Researchgate.net, or Academia.edu is one factor that contributes to the availability of at least one image from the article on Google Images (OR 2.99, 95% CI 1.20-7.45). This means that if the article is freely available online, there is 2.99 times the likelihood of finding an image from that article on Google Images. The odds ratio was also calculated for the following factors: color photographs (OR 1.09, 95% CI 0.39-3.07), photographs visible on journal landing page (OR 0.88, 95% CI 0.37-2.10), and pre- or postsurgery images of genitals (OR 0.62, 95% CI 0.23-1.44).

## Discussion

### Principal Findings

In this cross-sectional study, we sought to determine what percentage of medical images from trans-focused peer-reviewed journal articles published between 2008 and 2015 was available on Google Images. Thirty-five articles out of 94 (37%) included at least one photograph that was found on Google Images, and the likelihood was almost three times higher for articles that were openly available through open access or other Web-based platforms. In total, 20.3% (123/605) of clinical photographs were found on Google Images. Given that it is unlikely that patients consented to sharing their personal health information in these ways, this constitutes a risk to patient privacy. For research studies, this likely also poses a problem in relation to research ethics guidelines.

### Strengths and Limitations

To our knowledge, this is the first study to document the existence of medical photographs from peer-reviewed journals appearing in Google Images search results. One of its strengths is the systematic, documented approach to searches across a range of platforms including Google Scholar, PubMed, Researchgate.net, and Academia.edu.

At this time, there are no other studies with which to compare our results. This study included published clinical studies focused specifically on trans people. Given the emphasis on medical transition and gender affirming surgeries, it is likely that these publications include a higher number of genital, chest, or breast photographs than a random sample related to the overall population.

There are also other potential image databases we could have searched. In developing our search strategy, we tried several alternatives. However, it is important to note that some image search engines filter out results that are not family- or child-friendly. With the number of sensitive photographs in our dataset, it was important to select a search engine with the potential to clearly adjust these settings: Google Images provided this option (although it is unclear how many of the images may have been filtered out because they include pictures of genitals or breasts).

One of the challenges has been to understand the best way to search for images. After piloting several strategies, we focused on searching for the title of the article in quotation marks. This approach was more specific and led to a smaller number of search results than other search options. As technology develops, it will be possible to compare multiple search strategies and to search directly by image without using text. Google Image currently has this option, but we did not have the image files at the beginning of our searches in order to pursue this strategy.

Another difficulty is that the results of searches on Google Images are not static. This means that over time the results will shift, and medical photographs found during this search may not appear in the future. Similarly, images that were not found as part of this study could appear in future searches. Another challenge with this search is that sometimes images from one article in the dataset would appear in the results of a search for a different article. It seems that sometimes this was because of overlapping authors on the research teams, but at other times this appeared to be linked to the keywords (eg, phalloplasty or vaginoplasty). We did not include this information in our results, but this is an area for future attention.

When considering these challenges, it is important to keep the larger purpose of this study in mind. We were interested in determining what percentage of medical photographs from a sample of peer-reviewed journal articles were available on Google Images. While the specific results related to each photograph and article matter, the more important finding is the overall number of images we were able to find on Google Images and the implications for patients, clinicians, policy makers, and publishers.

It should also be noted that this sample was drawn from trans-focused publications. We have not yet tested whether the results would be similar for a general sample of clinical publications. However, given the methods used we have no reason to believe that the findings would be different. We plan to test this hypothesis in future studies.

### Implications for Patients, Clinicians, Policy Makers, and Publishers

In over a third of the articles in this dataset, at least one medical photograph was located on Google Images. Almost all of the images we searched for included graphic photographs of patient genitals, chests, or breasts. Many of the pictures were taken during surgery or included pre- and postsurgical images. A number included patient faces or other identifying features with no attempts at anonymization. Some photographs included full-body images of people who are deceased, at times displayed in ways that did not preserve human dignity.

For trans people, the ability to control access to information about their lives as people who are transgender, transsexual, or gender non-binary has concrete ramifications linked to disclosure. This includes decisions about personal health information including medical photographs. Trans people experience heightened rates of discrimination, harassment, and violence [16,17], particularly those who are visibly gender non-binary or whose trans identities become known to individuals in their lives who may not have been previously aware of this information [18]. There are a limited number of surgeons and other medical specialists who provide care to trans patients. Typically, within a state or geographic region there may be only one or two providers who specialize in performing specific types of gender affirming surgeries. In Canada for example, there is only one clinic in the country where vaginoplasties and phalloplasties are conducted. These circumstances contribute to heightened privacy and confidentiality concerns for trans patients. The limited number of surgeons means that patients can be identified more easily based on the combined information provided in medical photographs alongside descriptive case reports. While there may be value in the use of medical photographs for education, clinical care, and research, there are serious ethical issues to consider in relation to the visibility of medical images in public search results: these concerns are enhanced for trans individuals.

There are multiple intervention points to address this situation. The first strategy is to inform patients as part of the written informed consent process that their medical photograph(s) could appear in online image databases, including the search results from Google Images. In addition to current guidelines indicating that patients should be shown a copy of any photographs that will be published as part of case reports or other medical publications, they need to be aware of the potential for their photograph to become more widely available on the Internet. Current research with patients in the field of dermatology suggests that patients are more open to having their images shared between medical practitioners than via websites [29].

As part of informing patients, institutional policies for medical photography and guidelines for clinicians will also need to reflect the risk of medical photographs appearing openly online. Similarly, journal guidelines for the use of medical images should address these possibilities and any steps being taken to mediate these risks.

A second strategy is to determine how online image databases are accessing medical photographs from peer-reviewed publications. While this may be facilitated by open access publishing, there are articles that are not available through open access or other online websites where the photographs still appear in Google Images search results. In June 2017, Google changed its policies to state that it will remove “confidential, personal medical records of private people” from its search results [30]. While it is currently unclear whether this will affect photographs published in peer-reviewed journals, it would be helpful to have additional information about this shift in practice and whether other search engines will institute equivalent policies.

Another option is for journal publishers to take a different approach to the ways medical photographs are shared online. For example, some publishers have medical photographs available as PowerPoint files but accessible only behind their paywall. As part of the chain of stakeholders sharing patient medical information, publishers also have a responsibility to consider the implications of patient photographs being shared through massive searchable image repositories such as Google Images.

### Conclusions

The drive towards unrestricted open access suggests that the boundaries of the public domain between academic journals and search engines such as Google Images will become more porous. The public, the technology sector, and other stakeholders including clinicians, researchers, and policy makers all have stakes in the ways information travels across platforms. As Open Science [31] is increasingly emphasized, the need for critical perspectives is paramount [32]. Clinicians, researchers, and policy makers must respect patient autonomy, including attention to patient confidentiality, privacy, and informed consent. Medical photographs form part of the patient record and as such are subject to guidelines concerning the privacy of patient health information, regardless of the type of image and whether or not the patient is recognizable. In terms of copyright, these images may also be owned or co-owned by patients themselves. As part of research studies, medical photographs constitute part of the confidential data contributed by participants. Based on the impact of current practices, revisions to informed consent policies and guidelines are required. It is recommended that informed consent documents reflect the potential for medical photographs published in peer-reviewed journals to appear in image repositories such as Google Images search results. These new informed consent policies should be developed in collaboration with patients, with particular attention to images that may be published in online peer-reviewed publications or other online formats. In addition to the need for informed consent documents that detail all potential uses of medical photographs, it is recommended that journal publishers move to protect patient privacy by investigating technical options that would block the capture of medical images from their websites. Finally, the appearance of medical photographs in Google Images or other online search results after publication is also related to the technology and practices that online search engines use to capture images and create their image database, which are largely out of the control of manuscript authors and journal publishers. To address the root cause of this issue, search engines’ relevant policy and practice guidance should be revised.

## Appendix

Multimedia Appendix 1 Sample PubMed search strategy for original evidence map.

Multimedia Appendix 2 Full list of clinical studies with medical photographs.

## Abbreviations

IP: Internet protocol
LGBT: lesbian, gay, bisexual, or transgender
OR: odds ratio
PRISMA: Preferred Reporting Items for Systematic Reviews and Meta-Analyses
Trans: transgender, gender non-binary, and other gender diverse people
