# Supplementary material for: Open Availability of Patient Medical Photographs in Google Images Search Results: Cross-Sectional Study of Transgender Research
Source: J Med Internet Res. 2018 Feb 26;20(2):e70. doi: 10.2196/jmir.8787 (PMC5847816; doi:10.2196/jmir.8787)
Supplement: Multimedia Appendix 1 [file jmir_v20i2e70_app1.pdf]

Multimedia Appendix 1: Sample PubMed search strategy for original evidence map

#	Query
3	transsex*[TIAB] OR transex*[TIAB] OR transgender*[TIAB] OR travesti*[TIAB] OR transvesti*[TIAB] OR gender identity disorder*[TIAB] OR gender dysphori*[TIAB] OR two-spirit*[TIAB]
7	sex reassignment[TIAB] OR sexual reassignment[TIAB] OR gender reassignment[TIAB] OR gender confirmation[TIAB] OR sex change operation*[TIAB]
9	transman[TIAB] OR transmen[TIAB] OR "trans man"[TIAB] OR "trans men"[TIAB] OR "trans person"[TIAB] OR "trans persons"[TIAB] OR "trans people"[TIAB] OR "trans individual"[TIAB] OR "trans individuals"[TIAB] OR transwoman[TIAB] OR transwomen[TIAB] OR "trans woman"[TIAB] OR "trans women"[TIAB] OR "trans identity"[TIAB] OR "trans identities"[TIAB] OR "trans youth"[TIAB] OR "trans parent"[TIAB] OR "trans parenting"[TIAB] OR "trans parents"[TIAB] OR "trans elder"[TIAB] OR "trans elders"[TIAB] OR "trans senior"[TIAB] OR "trans seniors"[TIAB] OR "pregnant man"[TIAB] OR "pregnant men"[TIAB]
11	LGBT*[TIAB] OR GLBT*[TIAB] OR BLGT*[TIAB] OR BGLT*[TIAB] OR LBGT*[TIAB] OR GBLT*[TIAB]
13	transmasculin*[TIAB] OR transfeminin*[TIAB] OR genderqueer*[TIAB] OR gender queer*[TIAB] OR genderfluid*[TIAB] OR "gender fluid"[TIAB] OR gender varian*[TIAB] OR gender nonconform*[TIAB] OR autogynephil*[TIAB] OR gender identity clinic*[TIAB] OR gender identity service*[TIAB]
14	transphobi*[TIAB] OR cisgender*[TIAB] OR cisnorm*[TIAB] OR transfeminis*[TIAB] OR "trans feminist"[TIAB] OR "trans feminism"[TIAB] OR berdache*[TIAB] OR tranny[TIAB] OR eunuch[TIAB] OR "gender bender"[TIAB] OR bigender*[TIAB] OR cross-dress*[TIAB] OR co-gender[TIAB] OR third gender[TIAB] OR third sex[TIAB] OR gender binar*[TIAB] OR assigned female at birth[TIAB] OR assigned female at birth[TIAB] OR ladyboy*[TIAB] OR trigender[TIAB] OR drag king[TIAB] OR drag queen[TIAB]
25	harry benjamin[TIAB] OR HBIGDA[TIAB] OR WPATH[TIAB]
27	trans-sexual*[TIAB] OR trans-gender*[TIAB]

30	(gender[TIAB] AND trans[TIAB]) NOT ("fatty acid" OR "trans fat" OR "trans fats" OR "trans membrane" OR "trans pars plana vitrectomy" OR all-trans OR fatty OR rabbit OR rabbits OR rat OR rats OR sheep OR pig OR pigs OR bear OR bears OR dolphin OR dolphins OR Hb Baden OR trans-1 OR trans-2 OR trans-3 OR trans-3' OR trans-4 OR trans-7 OR trans-8 OR trans-9 OR trans-10 OR trans-11 OR trans-abdominal OR trans-acting OR trans-activation OR trans-anal OR trans-anastomotic OR trans-anorectal OR trans-aortic OR trans-apical OR trans-arterial OR trans-associations OR trans-atlantic OR Trans-bronchial OR trans-canal OR trans-canalicular OR trans-carveol OR trans-catheter OR trans-cerebellar OR trans-cervical OR trans-choroidal OR trans-cinnamaldehyde OR trans-CMF OR trans-conjunctival OR trans-corneal OR trans-cranial OR trans-cultural OR trans-cutaneous OR trans-dermal OR trans-diagnostic OR trans-disciplin* OR Trans-Dniester OR trans-endothelial OR trans-epidermal OR trans-epithelial OR trans-esophageal OR trans-factors OR trans-fascially OR trans-femoral OR transfemoral OR *transferase OR Transferrin OR trans-fistula OR trans-focal OR trans-gene OR trans-generation* OR trans-generic OR trans-genic OR transgenic OR trans-golgi OR trans-gulf OR trans-hernial OR Trans-Himalaya OR trans-historic* OR trans-iliac OR trans-illumination OR trans-impedance OR trans-imperial OR trans-infection OR trans-institution* OR trans-interactive OR trans-lamina OR trans-laryngeal OR trans-linalool OR trans-lycopene OR tran-sition OR translate OR translation* OR trans-luminal OR trans-mammary OR trans-mandibular OR trans-marrow OR trans-mediterranean* OR trans-membrane OR Trans-Mexican OR transmitral OR trans-muconic OR transmural OR transnation* OR trans-nation* OR trans-nonachlor OR trans-obturator OR trans-oesophageal OR trans-operative OR trans-oral OR trans-pacific OR trans-placental OR transplant OR transport OR trans-professional OR transpupillary OR trans-radial OR trans-region* OR trans-renal OR trans-ritual OR trans-sac OR trans-scleral OR trans-section* OR trans-sectoral OR transcription OR trans-signalling OR trans-situational OR trans-SOF OR trans-species OR trans-sphenoidal OR trans-sphincter* OR trans-stenotic OR trans-sternal OR trans-stimulatory OR trans-styloid OR trans-sylvian OR trans-tetrhydrocanabinol OR trans-theoretical OR trans-thoracic OR trans-tibial OR trans-tramadol OR trans-tricuspid OR trans-tympanic OR trans-umbilical OR trans-vitreal OR trans-zygomatic OR transsex* OR transgender* OR transex* OR "gender identity disorder" OR "gender dysphoric" OR "gender dysphoria" OR LGBT* OR cardiac OR aorta OR marine OR multiple sclerosis OR Tasman OR allele OR gene OR lung OR lungs)
32	gender minorit*[TIAB]
34	(gender divers*[TIAB] AND (sexual[TIAB] OR sexuality[TIAB] OR sexualities[TIAB])) NOT (board[TIAB] OR corporate[TIAB] OR corporation[TIAB] OR employment[TIAB] OR LGBT*[TIAB] OR transgender*[TIAB] OR transsex*[TIAB] OR transex*[TIAB] OR gender identity disorder[TIAB] OR gender dysphori*[TIAB])
36	gender identit*[TIAB] AND (sex[TIAB] OR sexual[TIAB] OR sexualit*[TIAB]) NOT (LGBT*[TIAB] OR transgender*[TIAB] OR transsex*[TIAB] OR transex*[TIAB] OR gender identity disorder[TIAB] OR gender dysphori*[TIAB] OR "disorders of sex development"[TIAB])

38	Hijra[TIAB] OR hirja[TIAB] OR kothi[TIAB] OR kathoey[TIAB] OR muxe[TIAB] OR "sworn virgin"[TIAB] OR fa-afafine[TIAB] OR fakafefine[TIAB] OR Nadleehi[TIAB] OR Fakaleiti[TIAB] OR Bakla[TIAB] OR Calabai[TIAB] OR calalai[TIAB] OR (waria[TIAB] NOT "Waria Valley") OR (Acault[TIAB] AND Myanmar) OR (Metis[TIAB] AND Nepal) OR Aravani[TIAB] OR Xanith[TIAB] OR Skoptsy[TIAB] OR mahuwahine[TIAB] OR mahu[TIAB]
42	Femminiello[TIAB] OR basivi[TIAB] OR Ninauposkitzipxpe[TIAB] OR Winkte[TIAB] OR Lhamana[TIAB] OR Dilbaa[TIAB] OR Alyha[TIAB] OR Hwame[TIAB] OR Muxhe[TIAB] OR Guevedoche[TIAB] OR machi-embra[TIAB] OR Quariwarmi[TIAB] OR Whakawahine[TIAB] OR Wakatane[TIAB] OR Bissu[TIAB] OR "Khwaja Saraa"[TIAB] OR Sekrata[TIAB] OR Mashoga[TIAB] OR Ashtime[TIAB] OR Burrnesha[TIAB] OR kocek[TIAB] OR (Mino[TIAB] AND Benin)
50	metoidioplast*[TIAB] OR phalloplast*[TIAB] NOT (deficiency OR augmentation OR congenital OR cadaver* OR aphallia OR penectomy OR "disorders of sex development" OR "disorder of sex development" OR DSD OR “androgen insensitivity syndrome” OR burn OR burns OR cancer OR cancers OR cancerous)
51	Vaginectomy[TIAB] NOT (“disorder of sex development” OR "disorder of sex development" OR DSD OR carcinoma OR carcinomas OR adenocarcinoma* OR congenital* OR “Mayer-Rokitansky-Küster-Hauser” OR “herlyn-werner-wunderlich” OR intersex* OR “vaginal agenesis” OR neovagina OR tumor OR tumors OR tumour* OR cancer OR cancers OR cancerous OR malignant* OR malignancy* OR oncolog* OR Mullerian OR dog OR dogs OR canine OR rat OR rats OR Colpocleisis OR prolapse)
52	(mastectom*[TIAB] OR chest reconstruction[TIAB]) NOT (tumour* OR tumor OR tumors OR "breast cancer" OR radiation OR chemotherapy OR mammogram OR mammography OR carcinoma* OR "breast reconstruction" OR malignant OR malignancy OR malignancies OR radiotherapy OR oncological OR thoracic OR oncology OR adipose OR liver* OR cancer OR cancers OR cancerous OR prophylactic OR diabetes OR diabetic* OR opioid OR gynecomastia* OR BRCA OR gene OR "breast augmentation" OR goat* OR dog OR dogs OR canine OR mice OR mouse OR rat OR rats OR "chronic kidney disease" OR "juvenile mammary hypertrophy" OR scoliosis OR biopsy OR congenital OR encephalopathy OR tamoxifen OR Gitelman OR rabbit* OR “periductal mastitis” OR “breast volume” OR “necrotizing fasciitis” OR “bariatric surgery” OR “breastfeeding” OR cow OR cows OR calf OR abdominoplasty OR mastoidectomy OR cadaver* OR Tasman)
53	(vaginoplast*[TIAB] OR neovagina[TIAB]) NOT (“disorders of sex development” OR "disorder of sex development" OR DSD OR congenital* OR “androgen insensitivity syndrome” OR intersex* OR muller* OR agenes* OR Mayer-Rokitansky-Kuster-Hauser OR MRKH OR “Bardet Biedl” OR “cloacal exstrophy” OR oncolog* OR sarcoma* OR carcinoma* OR cancer OR cancers OR cancerous OR burn* OR cat OR cats OR kitten OR calf)

54	(orchiectomy[TIAB] OR orchidectomy[TIAB] OR penectomy[TIAB]) NOT (prostate OR neoplasm* OR cancer OR cancers OR cancerous OR carcinom* OR oncolog* OR tumour OR tumor OR tumours OR tumors OR malignant OR malignancy OR malignancies OR benign OR seminoma OR torsion OR tortion OR undescended OR "androgen insensitivity syndrome" OR congenital* OR "disorders of sex development" OR "disorder of sex development" OR DSD OR hernia* OR vasculitis OR lymphoma* OR sarcoma* OR gangrene* OR cyst OR cysts OR aneurysm* OR mice OR mouse OR rat OR rats OR canine* OR deer OR pig OR pigs OR wether* OR "sea otter" OR cattle OR stallion* OR rabbit* OR hamster* OR cat OR cats OR chicken* OR monkey* OR bull OR turtle* OR animal* OR sheep OR tortoise* OR horse* OR dog OR dogs OR elephant* OR primate* OR hippopotamus OR veterinar* OR pig OR pigs OR buck* OR reptile* OR boar* OR lizard* OR bear*)
55	Genitoplasty[TIAB] NOT ("disorders of sex development" OR "disorder of sex development" OR DSD OR congenital* OR intersex* OR "androgen insensitivity syndrome")
56	(silicone[TIAB] AND trans[TIAB]) NOT (trans-fistula OR trans-tibial OR trans-choroidal OR trans-dermal OR trans-scleral OR trans-cutaneous OR trans-cranial OR trans-CMF OR "fatty acid" OR trans-gene OR tran-sition OR trans-1 OR trans-2 OR trans-3 OR trans-4 OR trans-7 OR trans-8 OR trans-9 OR trans-11 OR trans-membrane OR trans-impedance OR trans-epidermal OR all-trans OR trans-SOF OR trans-cinnamaldehyde OR trans-abdominal OR trans-sphincter* OR trans-carveol OR trans-obturator OR trans-anal OR trans-tympanic OR trans-aortic OR trans-mandibular OR trans-zygomatic OR rat OR rats OR rabbit OR rabbits OR sheep OR trans-stimulatory OR trans-endothelial OR trans-conjunctival OR trans-apical OR trans-vitreal OR trans-radial OR trans-sylvian OR trans-anorectal OR trans-femoral OR trans-canalicular OR trans-sac OR "trans pars plana vitrectomy" OR transpupillary OR transport OR trans-placental OR trans-mammary OR trans-illumination OR trans-arterial OR trans-catheter OR trans-renal OR trans-sternal OR trans-tetrhydrocanabinol)
57	(hair removal[TIAB] AND trans)
58	(electrolysis[TIAB] AND trans) NOT (trans-L OR trans-configuration OR trans-N OR trans-granular OR trans-position OR trans-3 OR electrochemical OR electrochemistry OR trans-form OR trans-bis OR trans-array OR chemistry OR rabbit OR rabbits OR glaucoma OR cochlear OR pacemaker OR eyelid)
59	facial feminization[TIAB] OR facial feminisation[TIAB]
60	tracheal shave[TIAB] OR chondrolaryngoplasty[TIAB] OR cricothyroidopexy[TIAB]
64	Transgendered persons[MESH] OR Transsexualism[MESH] OR Sex Reassignment Procedures[MESH] OR Sex Reassignment Surgery[MESH] OR Health Services for Transgendered Persons[MESH] OR Transvestism[MeSH]
66	(female to male[TIAB]) AND FTM[TIAB]
68	(male to female[TIAB]) AND MTF[TIAB]
