# Supplementary material for: Open Availability of Patient Medical Photographs in Google Images Search Results: Cross-Sectional Study of Transgender Research
Source: J Med Internet Res. 2018 Feb 26;20(2):e70. doi: 10.2196/jmir.8787 (PMC5847816; doi:10.2196/jmir.8787)
Supplement: Multimedia Appendix 2 [file jmir_v20i2e70_app2.pdf]

Multimedia Appendix 2: Full List of Clinical Studies with Medical Photographs

Adenuga, P., Summers, P., & Bergfeld, W. (2012). Hair regrowth in a male patient with extensive androgenetic alopecia on estrogen therapy. *Journal of the American Academy of Dermatology*, *67*(3), e121-e123.

Adelowo, A., Weber-LeBrun, E. E., & Young, S. B. (2009). Neovaginectomy following vaginoplasty in a male-to-female transgender patient: A case report and review of literature. *Journal of Pelvic Medicine and Surgery*, *15*(3), 101-104.

Ahlin, H. B., Kolby, L., Elander, A., & Selvaggi, G. (2014). Improved results after implementation of the Ghent algorithm for subcutaneous mastectomy in female-to-male transsexuals. *Journal of Plastic Surgery and Hand Surgery*, *48*(6), 362-367.

Alameddine, A. K., Alimov, V. K., Turner Jr., G. S., & Deaton, D. W. (2011). Surgical pitfalls of excising an intramyocardial lipoma. *Journal of Thoracic and Cardiovascular Surgery*, *141*(2), 592-594

Altman, K. (2012). Facial feminization surgery: Current state of the art. *International Journal of Oral and Maxillofacial Surgery*, *41*(8), 885-894.

Altomare, D. F., Scalera, I., Bettocchi, C., & Di Lena, M. (2013). Graciloplasty for recurrent recto-neovaginal fistula in a male-to-female transsexual. *Techniques in Coloproctology*, *17*(1), 107-109.

Amend, B., Seibold, J., Toomey, P., Stenzl, A., & Sievert, K. D. (2013). Surgical reconstruction for male-to-female sex reassignment. *European Urology*, *64*(1), 141-149.

Aminsharifi, A., Afsar, F., Jafari, M., & Tourchi, A. (2012). Removal of an entrapped large metallic dilator from the sigmoid neovagina in a male-to-female transsexual using a laparoscopic approach. *International Journal of Surgery Case Reports 3*(7), 266-268.

Antoszewski, B., Bratoś, R., Sitek, A., & Fijałkowska, M. (2012). Long-term results of breast reduction in female-to-male transsexuals. *Polski Przeglad Chirurgiczny/ Polish Journal of Surgery*, *84*(3), 144-151.

Atanasijević, T., Jovanović, A. A., Nikolić, S., Popović, V., & Jašović-Gašić, M. (2009). Accidental death due to complete autoerotic asphyxia associated with transvestic fetishism and anal self-stimulation-case report. *Psychiatria Danubina*, *21*(2), 246-251.

Berry, M. G., Curtis, R., & Davies, D. (2012). Female-to-male transgender chest reconstruction: A large consecutive, single-surgeon experience. *Journal of Plastic, Reconstructive & Aesthetic Surgery*, *65*(6), 711-719.

Bogliolo, S., Cassani, C., Babilonti, L., Gardella, B., Zanellini, F., Dominoni, M., … & Spinillo, A. (2014). Robotic single-site surgery for female-to-male transsexuals: Preliminary experience. *Scientific World Journal*, 674579. doi: 10.1155/2014/674579

Bucci, S., Mazzon, G., Liguori, G., Napoli, R., Pavan, N., Bormioli, S., … & Trombetta, C. (2014). Neovaginal prolapse in male-to-female transsexuals: An 18-year-long experience. *BioMed Research International*, 240761. doi: 10.1155/2014/240761

Camp, S., Cartwright, P., & Siddiqi, F. (2011). The prefabricated gracilis muscle flap with full-thickness skin graft and delay for urethral channel reconstruction. *Annals of Plastic Surgery*, *67*(1), 59-61.

Capitán, L., Simon, D., Kaye, K., & Tenorio, T. (2014). Facial feminization surgery: The forehead. Surgical techniques and analysis of results. *Plastic and Reconstructive Surgery, 134*(4), 609-619.

Carella, S., Romanzi, A., Ciotti, M., & Onesti, M. G. (2013). Skin ulcer: A long-term complication after massive liquid silicone oil infiltration. *Aesthetic Plastic Surgery, 37*(6), 1220-1224.

Caricato, M., Ausania, F., Marangi, G. F., Cipollone, I., Flammia, G., Persichetti, P., ... & Coppola, R. (2009). Surgical treatment of locally advanced anal cancer after male-to-female sex reassignment surgery. *World Journal of Gastroenterology: WJG, 15*(23), 2918-2919.

Cavadas, P. C. (2008). Secondary free fibular flap for providing rigidity in a radial forearm phalloplasty. *Plastic and Reconstructive Surgery, 122*(2), 101e-102e.

Chen, M., Yalamanchili, C., Hamous, J., Piskun, M. A., & Weis, B. (2008). Acute inflammatory response of the male breasts secondary to self-injection of petroleum jelly: A case report. *Southern Medical Journal, 101*(4), 422-424.

Cho, S. W., & Jin, H. R. (2012). Feminization of the forehead in a transgender: Frontal sinus reshaping combined with brow lift and hairline lowering. *Aesthetic Plastic Surgery, 36*(5), 1207-1210.

Cohen, B., Parker, D., Lu, C. T., & Strahan, A. (2011). An unusual case of caecal volvulus. *ANZ Journal of Surgery, 81*(12), 944-945.

Cornetta, S., Addante, A., Zotti, F., & Dell'Erba, A. (2009). Unusual death of a transvestite: Identification of crime weapon and survival time. *Journal of Forensic Sciences*, *54*(5), 1149-1151.

Cregten-Escobar, P., Bouman, M. B., Buncamper, M. E., & Mullender, M. G. (2012). Subcutaneous mastectomy in female-to-male transsexuals: A retrospective cohort-analysis of 202 patients. *Journal of Sexual Medicine*, *9*(12), 3148-3153.

de Matos, L. L., Lopes, L. S., de Andrade Saggiomo, J. V., Wroclawski, E. R., & Junior, M. P. F. (2009). Acute urinary retention as a late complication of subcutaneous liquid silicone injection: A case report. *Einstein (Säo Paulo)*, *7*(4), 509-511.

Dempf, R., & Eckert, A. W. (2010). Contouring the forehead and rhinoplasty in the feminization of the face in male-to-female transsexuals. *Journal of Cranio-Maxillofacial Surgery*, *38*(6), 416-422.

Dessy, L. A., Mazzocchi, M., Buccheri, E. M., & Figus, A. (2009). The ring stitches, a useful method to manage vaginal stent in male-to-female transsexuals. *Journal of Plastic, Reconstructive & Aesthetic Surgery*, *62*(3), 409-410.

Dessy, L. A., Mazzocchi, M., Corrias, F., Ceccarelli, S., Marchese, C., & Scuderi, N. (2014). The use of cultured autologous oral epithelial cells for vaginoplasty in male-to-female transsexuals: A feasibility, safety, and advantageousness clinical pilot study. *Plastic and Reconstructive Surgery*, *133*(1), 158-161.

Dickerson, E. M., Jones, P., Wilkins, D., Regnier, J., & Prahlow, J. A. (2013). Complicated suicide versus autoeroticism?: A case involving multiple drugs and a porta-potty. *American Journal of Forensic Medicine and Pathology*, *34*(1), 29-33.

Djordjevic, M. L., & Bizic, M. R. (2013). Comparison of two different methods for urethral lengthening in female to male (metoidioplasty) surgery. *Journal of Sexual Medicine*, *10*(5), 1431-1438.

Djordjevic, M. L., Bizic, M., Stanojevic, D., Bumbasirevic, M., Kojovic, V., Majstorovic, M., … & Perovic, S. V. (2009). Urethral lengthening in metoidioplasty (female-to-male sex reassignment surgery) by combined buccal mucosa graft and labia minora flap. *Urology*, *74*(2), 349-353.

Djordjevic, M. L., Stanojevic, D., Bizic, M., Kojovic, V., Majstorovic, M., Vujovic, S., … & Perovic, S. V. (2009). Metoidioplasty as a single stage sex reassignment surgery in female transsexuals: Belgrade experience. *Journal of Sexual Medicine*, *6*(5), 1306-1313.

El Muayed, M., Costas, A., & Pick, A. (2009). 1, 25-dihydroxyvitamin D-mediated hypercalcemia in oleogranulomatous mastitis (paraffinoma), ameliorated by glucocorticoid administration. *Endocrine Practice*, *16*(1), 102-106.

Garaffa, G., Christopher, N. A., & Ralph, D. J. (2010). Total phallic reconstruction in female-to-male transsexuals. *European Urology*, *57*(4), 715-722.

Garaffa, G., Ralph, D. J., & Christopher, N. (2010). Total urethral construction with the radial artery-based forearm free flap in the transsexual. *BJU International*, *106*(8), 1206-1210.

Garcia, M. M., Christopher, N. A., De Luca, F., Spilotros, M., & Ralph, D. J. (2014). Overall satisfaction, sexual function, and the durability of neophallus dimensions following staged female to male genital gender confirming surgery: The Institute of Urology, London UK experience. *Translational Andrology and Urology*, *3*(2), 156-162.

Hamdan, A. (2012). Cricothyroid approximation using a silastic sheath: A new approach. *Middle East Journal of Anesthesiology, 21*(6), 909-912.

Hasegawa, K., Namba, Y., & Kimata, Y. (2013). Phalloplasty with an innervated island pedicled anterolateral thigh flap in a female-to-male transsexual. *Acta Medica Okayama*, *67*(5), 325-331.

Hoebeke, P. B., Decaestecker, K., Beysens, M., Opdenakker, Y., Lumen, N., & Monstrey, S. M. (2010). Erectile implants in female-to-male transsexuals: Our experience in 129 patients. *European Urology, 57*(2), 334-341.

Jarolim, L., Sedy, J., Schmidt, M., Nanka, O., Foltán, R., & Kawaciuk, I. (2009). Gender reassignment surgery in male-to-female transsexualism: A retrospective 3-month follow-up study with anatomical remarks. *Journal of Sexual Medicine*, *6*(6), 1635-1644.

Kannan, R. Y., Sankar, T. K., & Ward, D. J. (2010). The disaster of DIY breast augmentation. *Journal of Plastic, Reconstructive & Aesthetic Surgery, 63*(1), e100-e101.

Kim, S. K., Kim, T. H., Yang, J. I., Kim, M. H., Kim, M. S., & Lee, K. C. (2012). The etiology and treatment of the softened phallus after the radial forearm osteocutaneous free flap phalloplasty. *Archives of Plastic Surgery, 39*(4), 390-396.

Kim, S. K., Lee, K. C., Kwon, Y. S., & Cha, B. H. (2009). Phalloplasty using radial forearm osteocutaneous free flaps in female-to-male transsexuals. *Journal of Plastic, Reconstructive & Aesthetic Surgery, 62*(3), 309-317.

Kim, S. K., Moon, J. B., Heo, J., Kwon, Y. S., & Lee, K. C. (2010). A new method of urethroplasty for prevention of fistula in female-to-male gender reassignment surgery. *Annals of Plastic Surgery, 64*(6), 759-764.

Large, M. C., Gottlieb, L. J., Wille, M. A., DeWolfe, M., & Bales, G. T. (2009). Novel technique for proximal anchoring of penile prostheses in female-to-male transsexual. *Urology, 74*(2), 419-421.

Lee, K. C., Huang, C. Y., & Wang, P. H. (2012). Parasitic peritoneal leiomyomatosis mimicking intra-abdominal abscess with hematoma. *Taiwanese Journal of Obstetrics and Gynecology, 51*(1), 115-116.

Leyngold, M. M., & Rivera-Serrano, C. M. (2014). Microvascular penile replantation utilizing the deep inferior epigastric vessels. *Journal of Reconstructive Microsurgery, 30*(08), 581-584.

Lin, C. T., & Chen, L. W. (2009). Using a free thoracodorsal artery perforator flap for phallic reconstruction–A report of surgical technique. *Journal of Plastic, Reconstructive & Aesthetic Surgery, 62*(3), 402-408.

Lin, Y. C., Lin, W. C., & Hsu, J. M. (2013). Urethral stricture in male-to-female transsexual patients–Report of two cases. *Formosan Journal of Surgery, 46*(5), 173-175.

Majdak-Paredes, E. J., Shafighi, M., & Fatah, F. (2009). Unilateral hypoplastic breast in a male-to-female transsexual with Poland syndrome after gender reassignment–reconstructive considerations. *Journal of Plastic, Reconstructive & Aesthetic Surgery, 62*(3), 398-401.

Matsuki, S., Kusatake, K., Hein, K. Z., Anraku, K., & Morita, E. (2015). Condylomata acuminata in the neovagina after male-to-female reassignment treated with CO2 laser and imiquimod. *International Journal of STD & AIDS*, *26*(7), 509-511.

Mihm, L. B., Swetman, G., Boh, E. E., Wang, A., & Witzig, R. (2010). Patient with AIDS and acute circinate skin eruptions. *Clinical Infectious Diseases*, *51*(8), 980-982.

Monstrey, S. J., Ceulemans, P., & Hoebeke, P. (2011). Sex reassignment surgery in the female-to-male transsexual. *Seminars in Plastic Surgery*, *25*(3), 229-244.

Monstrey, S., Hoebeke, P., Selvaggi, G., Ceulemans, P., Van Landuyt, K., Blondeel, P., ... & De Cuypere, G. (2009). Penile reconstruction: Is the radial forearm flap really the standard technique? *Plastic and Reconstructive Surgery*, *124*(2), 510-518.

Monstrey, S., Selvaggi, G., Ceulemans, P., Van Landuyt, K., Bowman, C., Blondeel, P., ... & De Cuypere, G. (2008). Chest-wall contouring surgery in female-to-male transsexuals: A new algorithm. *Plastic and Reconstructive Surgery*, *121*(3), 849-859.

Muccino, E., Gentile, G., Marchesi, M., & Zoja, R. (2014). The homicide of a transgender by an ante-mortem “incaprettamento”. *Romanian Journal of Legal Medicine*, *22*(3), 157-160.

Murty, O. P. (2010). Male-to-female transsexual on estrogen: Sudden death due to pulmonary thromboembolism. *Journal of Forensic Medicine and Toxicology*, *27*(1), 27-34.

Namba, Y., Sugiyama, N., Yamashita, S., Tokuyama, E., Hasegawa, K., & Kimata, Y. (2008). Phantom erectile penis after sex reassignment surgery. *Acta Medica Okayama*, *62*(3), 213-216.

Namba, Y., Watanabe, T., & Kimata, Y. (2009). Mastectomy in female-to-male transsexuals. *Acta Medica Okayama*, *63*(5), 243-247.

Neto, R., Hintz, F., Krege, S., Rübben, H., & Vom Dorp, F. (2012). Gender reassignment surgery-a 13 year review of surgical outcomes. *International Brazilian Journal of Urology*, *38*(1), 97-107.

Papadopulos, N. A., Schaff, J., & Biemer, E. (2008). The use of free prelaminated and sensate osteofasciocutaneous fibular flap in phalloplasty. *Injury*, *39*(3), 62-67.

Raigosa, M., Avvedimento, S., & Fontdevila, J. (2013). Self-made compressive dressing for vaginoplasty. *Aesthetic Plastic Surgery*, *37*(4), 844-845.

Ranno, R., Vesely, J., Hyza, P., Stupka, I., Justan, I., Dvorak, Z., ... & Ranno, S. (2008). Neophalloplasty with a reinnervated latissimus dorsi free flap: A functional study of a novel technique. *Scripta Medica (BRNO)*, *81*(1), 1-22.

Reed, H. M. (2011). Aesthetic and functional male to female genital and perineal surgery: Feminizing vaginoplasty. *Seminars in Plastic Surgery*, *25*(2), 163-174.

Remacle, M., Matar, N., Morsomme, D., Veduyckt, I., & Lawson, G. (2011). Glottoplasty for male-to-female transsexualism: Voice results. *Journal of Voice*, *25*(1), 120-123.

Roerink, S., Marsman, D., van Bon, A., & Netea-Maier, R. (2014). A missed diagnosis of acromegaly during a female-to-male gender transition. *Archives of Sexual Behavior*, *43*(6), 1199-1201.

Rubino, C., Figus, A., Dessy, L. A., Alei, G., Mazzocchi, M., Trignano, E., & Scuderi, N. (2009). Innervated island pedicled anterolateral thigh flap for neo-phallic

reconstruction in female-to-male transsexuals. *Journal of Plastic, Reconstructive & Aesthetic Surgery*, *62*(3), e45-e49.

Schaff, J., & Papadopulos, N. A. (2009). A new protocol for complete phalloplasty with free sensate and prelaminated osteofasciocutaneous flaps: Experience in 37 patients. *Microsurgery*, *29*(5), 413-419.

Schenck, T. L., Holzbach, T., Zantl, N., Schuhmacher, C., Vogel, M., Seidl, S., ... & Giunta, R. E. (2010). Vaginal carcinoma in a female‐to‐male transsexual. *Journal of Sexual Medicine*, *7*(8), 2899-2902.

Schmidt, M., Grohmann, M., & Huemer, G. M. (2015). Pedicled superficial inferior epigastric artery perforator flap for salvage of failed metoidioplasty in female-to-male transsexuals. *Microsurgery*, *35*(5), 403-406.

Selvaggi, G., Branemark, R., Elander, A., Liden, M., & Stalfors, J. (2015). Titanium-bone-anchored penile epithesis: Preoperative planning and immediate postoperative results. *Journal of Plastic Surgery and Hand Surgery*, *49*(1), 40-44.

Selvaggi, G., Hoebeke, P., Ceulemans, P., Hamdi, M., Van Landuyt, K., Blondeel, P., ... & Monstrey, S. (2009). Scrotal reconstruction in female-to-male transsexuals: A novel scrotoplasty. *Plastic and Reconstructive Surgery*, *123*(6), 1710-1718.

Shams, M. G., & Motamedi, M. H. K. (2009). Case report: Feminizing the male face. *Eplasty*, *9*, 8-14.

Shimamura, Y., Fujikawa, A., Kubota, K., Ishii, N., Fujita, Y., & Ohta, K. (2015). Perforation of the neovagina in a male-to-female transsexual: A case report. *Journal of Medical Case Reports*, *9*(24). doi: 10.1186/1752-1947-9-24

Skugarevsky, O., Ehrlich, E., & Sheleg, S. (2011). Accidental strangulation resulted from hypoxyphilia associated with multiple paraphilias and substance abuse: A psychological autopsy case report. *Romanian Journal of Legal Medicine*, *19*(4), 249-252.

Soli, M., Brunocilla, E., Bertaccini, A., Palmieri, F., Barbieri, B., & Martorana, G. (2008). Male to female gender reassignment: Modified surgical technique for creating the neoclitoris and mons veneris. *Journal of Sexual Medicine*, *5*(1), 210-216.

Šolinc, M., Kosutic, D., Stritar, A., Planinsek, F., Mihelič, M., & Lukanovič, R. (2009). Preexpanded radial forearm free flap for one-stage total penile reconstruction in female-to-male transsexuals. *Journal of Reconstructive Microsurgery*, *25*(6), 395-398.

Song, C., Wong, M., Wong, C. H., & Ong, Y. S. (2011). Modifications of the radial forearm flap phalloplasty for female-to-male gender reassignment. *Journal of Reconstructive Microsurgery*, *27*(2), 115-120.

Sukumaran, S., Moran, P. A., & Makar, A. (2009). An unusual cause of vaginal discharge following gender reassignment. *International Urogynecology Journal*, *20*(2), 253-254.

Takamatsu, A., & Harashina, T. (2009). Labial ring flap: A new flap for metaidoioplasty in female-to-male transsexuals. *Journal of Plastic, Reconstructive & Aesthetic Surgery*, *62*(3), 318-325.

Tayade, P. J. (2011). Transsexualism. International Journal of Medical Toxicology & Legal Medicine, 13(4), 24-29.

Tchang, L. A., Largo, R. D., Babst, D., Wettstein, R., Haug, M. D., Kalbermatten, D. F., & Schaefer, D. J. (2014). Second free radial forearm flap for urethral reconstruction

after partial flap necrosis of tube-in-tube phalloplasty with radial forearm flap: A report of two cases. *Microsurgery*, *34*(1), 58-63.

Thione, A., Cavadas, P. C., & Carballeira, A. (2014). Urethra reconstruction with a prelaminated pedicled anterolateral thigh flap: A case report. *Annals of Plastic Surgery*, *72*(6), 695-697.

Tourbach, S. A., Hunter-Smith, D., & Morrison, W. A. (2011). Long anterior urethral reconstruction using a jejunal free flap. *Journal of Plastic Surgery and Hand Surgery*, *45*(1), 54-56.

Van Caenegem, E., Verhaeghe, E., Taes, Y., Wierckx, K., Toye, K., Goemaere, S., ... & T'Sjoen, G. (2013). Long-term evaluation of donor-site morbidity after radial forearm flap phalloplasty for transsexual men. *Journal of Sexual Medicine*, *10*(6), 1644-1651.

van der Sluis, W. B., Bouman, M. B., Gijs, L., & van Bodegraven, A. A. (2015). Gonorrhoea of the sigmoid neovagina in a male-to-female transgender. *International journal of STD & AIDS*, *26*(8), 595-598.

Vigneswaran, N., Lim, J., Lee, H. J., Ong, W. C., Rasheed, M. Z., & Lim, T. C. (2013). A novel technique with aesthetic considerations in female-to-male transsexuals nipple areola complex reconstruction. *Journal of Plastic, Reconstructive & Aesthetic Surgery*, *66*(12), 1805-1807.

Visnyei, K., Samuel, M., Heacock, L., & Cortes, J. A. (2014). Hypercalcemia in a male-to-female transgender patient after body contouring injections: A case report. *Journal of Medical Case Reports*, *8*(71). doi: 10.1186/1752-1947-8-71

Vukadinovic, V., Stojanovic, B., Majstorovic, M., & Milosevic, A. (2014). The role of clitoral anatomy in female to male sex reassignment surgery. *The Scientific World Journal*, 438378. doi: 10.1155/2014/437378

Wagner, S., Greco, F., Hoda, M. R., Inferrera, A., Lupo, A., Hamza, A., & Fornara, P. (2010). Male-to-female transsexualism: Technique, results and 3-year follow-up in 50 patients. *Urologia Internationalis*, *84*(3), 330-333.

Weigert, R., Frison, E., Sessiecq, Q., Al Mutairi, K., & Casoli, V. (2013). Patient satisfaction with breasts and psychosocial, sexual, and physical well-being after breast augmentation in male-to-female transsexuals. *Plastic and Reconstructive Surgery*, *132*(6), 1421-1429.

Weyers, S., De Sutter, P., Hoebeke, S., Monstrey, G., Sjoen, G. T., Verstraelen, H., & Gerris, J. (2010). Gynaecological aspects of the treatment and follow-up of transsexual men and women. *Facts*, *Views & Vision in ObGyn*, *2*(1), 35-54.

Wolter, A., Diedrichson, J., Scholz, T., Arens-Landwehr, A., & Liebau, J. (2015). Sexual reassignment surgery in female-to-male transsexuals: An algorithm for subcutaneous mastectomy. *Journal of Plastic*, *Reconstructive & Aesthetic Surgery*, *68*(2), 184-191.

Yang, C., Liu, S., Xu, K., Xiang, Q., Yang, S., & Zhang, X. (2009). Condylomata gigantea in a male transsexual. *International Journal of STD & AIDS*, *20*(3), 211-212.
